# Newborn and child health national and provincial clinical practice guidelines in South Africa, Nigeria and Malawi: a scoping review

**DOI:** 10.1186/s12913-024-10682-0

**Published:** 2024-02-19

**Authors:** Mashudu Mthethwa, Nyanyiwe Masingi Mbeye, Emmanuel Effa, Dachi Arikpo, Ntombifuthi Blose, Amanda Brand, Moriam Chibuzor, Roselyn Chipojola, Solange Durao, Ekpereonne Esu, Idriss Ibrahim Kallon, Gertrude Kunje, Suzgika Lakudzala, Celeste Naude, Trudy D. Leong, Simon Lewin, Denny Mabetha, Michael McCaul, Martin Meremikwu, Per Olav Vandvik, Tamara Kredo

**Affiliations:** 1 Health Systems Research Unit, South African Medical Research Council, Cape Town, South Africa; 2 Evidence Informed Decision-Making Centre, Department of Community and Environmental Health, School of Global and Public Health, Kamuzu University of Health Sciences, Lilongwe, Malawi; 3 Cochrane Nigeria, University of Calabar Teaching Hospital, Calabar, Nigeria; 4 Centre for Evidence-Based Health Care, Division of Epidemiology and Biostatistics, Department of Global Health, Stellenbosch University, Cape Town, South Africa; 5 Department of Health Sciences Ålesund, Norwegian University of Science and Technology (NTNU), Trondheim, Norway; 6 Department of Paediatrics, University of Calabar, Calabar, Nigeria; 7 MAGIC Evidence Ecosystem Foundation, Oslo, Norway; 8 Department of Medicine, Lovisenberg Diaconal Hospital, Oslo, Norway; 9 Division of Clinical Pharmacology, Department of Medicine, Faculty of Medicine and Health Sciences, Stellenbosch University, Cape Town, South Africa; 10 Department of Internal Medicine, University of Calabar, Calabar, Nigeria

**Keywords:** Clinical practice guidelines, Newborn and child health, Nigeria, South Africa, Malawi, Scoping review, Quality appraisal, GRADE

## Abstract

**Background:**

Low and middle-income countries remain disproportionately affected by high rates of child mortality. Clinical practice guidelines are essential clinical tools supporting implementation of effective, safe, and cost-effective healthcare. High-quality evidence-based guidelines play a key role in improving clinical management to impact child mortality. We aimed to identify and assess the quality of guidelines for newborn and child health published in South Africa, Nigeria and Malawi in the last 5 years (2017–2022).

**Methods:**

We searched relevant websites (June–July 2022), for publicly available national and subnational de novo or adapted guidelines, addressing newborn and child health in the three countries. Pairs of reviewers independently extracted information from eligible guidelines (scope, topic, target population and users, responsible developers, stakeholder consultation process, adaptation description, assessment of evidence certainty). We appraised guideline quality using the Appraisal of Guidelines for Research & Evaluation (AGREE II) instrument.

**Results:**

We identified 40-guidelines from the three countries. Of these, 8/40 reported being adopted from a parent guideline. More guidelines (*n* = 19) provided guidance on communicable diseases than on non-communicable diseases (*n* = 8). Guidelines were most often developed by national health ministries (*n* = 30) and professional societies (*n* = 14). Eighteen guidelines reported on stakeholder consultation; with Nigeria (10/11) and Malawi (3/6) faring better than South Africa (5/23) in reporting this activity. The Grading of Recommendations, Assessment, Development, and Evaluations (GRADE) approach was used in 1/7 guidelines that reported assessing certainty of evidence. Overall guidelines scored well on two AGREE II domains: scope and purpose median (IQR) score 68% (IQR 47–83), and clarity of presentation 81% (67–94). Domains critical for ensuring credible guidance scored below 20%: rigour of development 11% (4–32) and editorial independence 6% (0–27).

**Conclusion:**

National ministries and professional societies drive guideline activities in Malawi, Nigeria and South Arica. However, the methods and reporting do not adhere to global standards. We found low AGREE II scores for rigour of guideline development and editorial independence and limited use of GRADE or adaptation methods. This undermines the credibility of available guidelines to support evidence-informed care. Our findings highlight the importance of ongoing efforts to strengthen partnerships, capacity, and support for guideline development.

**Supplementary Information:**

The online version contains supplementary material available at 10.1186/s12913-024-10682-0.

## Introduction

Progress in reducing infant and child mortality has been made globally, with steady declines in under-five mortality witnessed in some sub-Saharan Africa (SSA) countries over the last decade (2009–2020); gains that are partly due to the successful implementation of antiretroviral therapy (ART) programmes [1, 2]. Despite these successes, most low- and middle-income countries (LMICs) remain disproportionately affected by high rates of child mortality [3]. About 80% of the five million deaths that occurred in children under five in 2020 occurred in SSA and South Asia [3]. Children born in SSA are 14 times more likely to die before reaching the age of five compared to children in high-income regions such as Europe and North America [3]. Mortality rates in SSA are predominantly driven by factors such as health inequity, poverty, poor health systems and poor nutrition [4, 5]. Furthermore, negative economic and health system impacts of the coronavirus 2019 (COVID-19) pandemic have skewed child mortality further towards poor and vulnerable populations [3, 6]. Poverty-related diseases including human immunodeficiency virus (HIV), pneumonia, diarrhoea, and malaria remain among the leading causes of death in children under five years in LMICs [3]. Most SSA countries are at risk of not meeting the Sustainable Development Goals (SDG) targets of reducing neonatal and under-five mortality rates to 12 per 1000 live births and 25 per 1000, respectively, by 2030 [7].

Newborn and child survival is often associated with high standards of healthcare including neonatal and obstetric care, as well as the socio-economic development of populations [8, 9]. However, in countries where inequity is high, or in those with fragmented health systems, the survival scale is often tipped in favour of well-off individuals with the capacity to sustain out-of-pocket expenses and private health insurance [9]. Hence, most SSA countries acknowledge the significance and role of Universal Health Care (UHC) in reaching the health SDG targets. The World Health Organization (WHO) maintains that health systems in low-resource settings where laboratory and radiology diagnostic services are limited or non-existent may benefit from using evidence-based algorithms and guidelines to determine the management for certain conditions [1]. Furthermore, guidelines may be instrumental for countries in the quest to achieve UHC as they inform the use of appropriate and effective health services to make the best use of scarce resources [2].

Clinical practice guidelines (referred to as guidelines for short in this manuscript) are “statements that include recommendations intended to optimise patient care that are informed by a systematic review of evidence and an assessment of the benefits and harms of alternative care options” [10]. Therefore, guidelines bridge the gap between research evidence and clinical practice, allowing the integration of the best scientific evidence with clinical expertise – as well as patients values and preferences, when making healthcare decisions [11]. Well-formulated guidelines are essential in standardising clinical decision-making, minimising error and wastage of health resources, and informing quality of care and funding decisions [10].

Key aspects of best-practice guidelines development methods include transparency and good governance, use of rigorously collected and synthesised research evidence, contextualisation, and appropriate methods for assessing certainty of evidence such as Grading of Recommendations Assessment, Development and Evaluation (GRADE) for reviews and guidelines [10, 12]. International organisations including the WHO, the Guideline International Network (GIN), the Institute of Medicine and the National Institute for Health and Care Excellence (NICE) have published standards for high-quality de novo (new) guideline development, adaptation, and implementation [13–15]. However, these resources are not consistently adopted by guideline developers, leading to the development in some settings of guidelines that do not meet international methodological standards. This aligns with the findings of a recent study which reported that 66% of the randomly sampled and assessed global guidelines (for any condition) were developed using non-systematic approaches to evidence synthesis [16].

To understand the landscape of newborn and child health guidelines in South Africa, Nigeria, and Malawi, this scoping review aimed to i) identify publicly available national and subnational guidelines for newborn and child health, ii) describe the scope of these guidelines, including methods used, and iii) appraise the quality and reporting standards of these guidelines. This study is part of a larger project, Global Evidence – Local Adaptation (GELA) [17], which aims to increase researchers and decision makers’ capacity to use global research evidence to develop locally relevant guidelines for newborn and child health. The GELA project aims to support decision makers in South Africa, Nigeria, and Malawi, and to build on and add value to the large-scale programme of global child health guideline development led by the WHO with adaptation and implementation led by the WHO Regional Office for Africa (Afro) and national ministries.

## Methods

We conducted a scoping review using a pre-defined protocol in accordance with the Arksey and O’Malley methodological approach [18] and reported the results according to the Preferred Reporting Items for Systematic reviews and Meta-Analyses extension for Scoping Reviews (PRISMA-ScR) checklist [18]. The protocol was developed by authors from South Africa, Nigeria, and Malawi, and implemented with teams in each country. The data from each country were then combined to create overall findings.

### Eligibility criteria

We included publicly available national and subnational level de novo, adapted and adopted guidelines, published between 2017 and 2022 in English or local languages of the respective countries. Guidelines were defined as documents that provide evidence-based clinical guidance and actionable recommendations for individual care. Guidelines were considered eligible if they provided guidance and recommendations for newborn and child health from birth to 12 years of age.

Guidelines were excluded if they included adolescents and adults only, were published before 2017, were not developed for South Africa, Nigeria, or Malawi, exclusively provided recommendations for health system or services delivery or implementation interventions or strategies (rather than clinical interventions) or had been replaced by newer or updated versions.

### Identification of guidelines

Search terms and information sources are presented in Supplementary file 1 (S1). Searches were conducted between June and July 2022 with no language restrictions. Each country team searched various sources including Google, Google scholar, respective Ministry of Health websites and policy document repositories, paediatric association websites, guideline clearing houses, GIN Library, Emergency Care Research Institute, Pan African Health Organization’s International Database of GRADE Guidelines], and relevant national journals with a focus on the content areas of interest to the landscape analysis (e.g. The Nigerian Journal of Paediatrics and the Tropical Journal of Obstetrics and Gynaecology which are the Journals of the Paediatric Association of Nigeria and Society of Gynaecology and Obstetrics of Nigeria respectively) using predefined and relevant key terms. We also contacted experts, including Ministry of Health representatives and clinicians, in each country to identify the most recently published versions of guidelines that are eligible, but are not found or published online. The experts were identified through policymaker and professional society networks that we have built through this project and previous guideline work. Although not all experts responded to our request, several did, providing suggestions for additional guidelines.

### Guideline selection

Pairs of authors independently screened titles and abstracts, and then full texts of retrieved documents, against the eligibility criteria. Any discrepancies were resolved through discussion or by involving a third author, if needed.

### Data extraction

One author independently extracted data from the included guidelines using a piloted Microsoft Excel data extraction sheet, with a second author verifying the data. Discrepancies were resolved through discussion. We extracted the following data: title; year of publication; topic or condition addressed by guideline; health care scope (e.g., prevention, treatment, diagnosis); target population; and target users. We also extracted data on whether stakeholders were consulted, whether the guideline was externally reviewed, and whether certainty of evidence was assessed; as well as descriptions of methods for guideline adaptation or adoption, and for assessing guideline applicability (costs associated with implementation, feasibility etc.).

### Critical appraisal: AGREE instrument

Pairs of reviewers independently assessed the quality and reporting standards of included guidelines using the online Appraisal of Guidelines for Research and Evaluation (AGREE II) instrument [19–21]. This instrument contains 23 items organized into six domains: scope and purpose, stakeholder involvement, rigor of development, clarity of presentation, applicability, and editorial independence. Each item was rated on a 7-point scale (1- strongly disagree to 7-strongly agree). The online AGREE II instrument automatically generates scores at the end of the guideline appraisal [21]. The standardized scores for individual AGREE domains range from 0 to 100% and are calculated using the following formula: (Obtained score – Minimum possible score) / (Maximum possible score – Minimum possible score)∗100%. The overall score was generated by making a judgement on the overall quality of the guideline while considering the domain scores. Any discrepancies in scoring were resolved by consensus or through consulting a third reviewer, where necessary. If a guideline explicitly stated that it was adopted or adapted, the quality of the parent guideline was appraised to understand the methods used.

### Data analysis

We analysed data using descriptive statistics, such as counts and proportions of characteristics of guidelines, using STATA 17 (StataCorp LLC, USA) and Microsoft Excel 2018. AGREE II scores were assessed for normality using the Shapiro-Wilk test and through visual inspection of box plots. As data were non-normally distributed, we reported medians with interquartile ranges. Extracted data were presented as counts and proportions.

## Results

### Results of the search

**Fig. 1:**
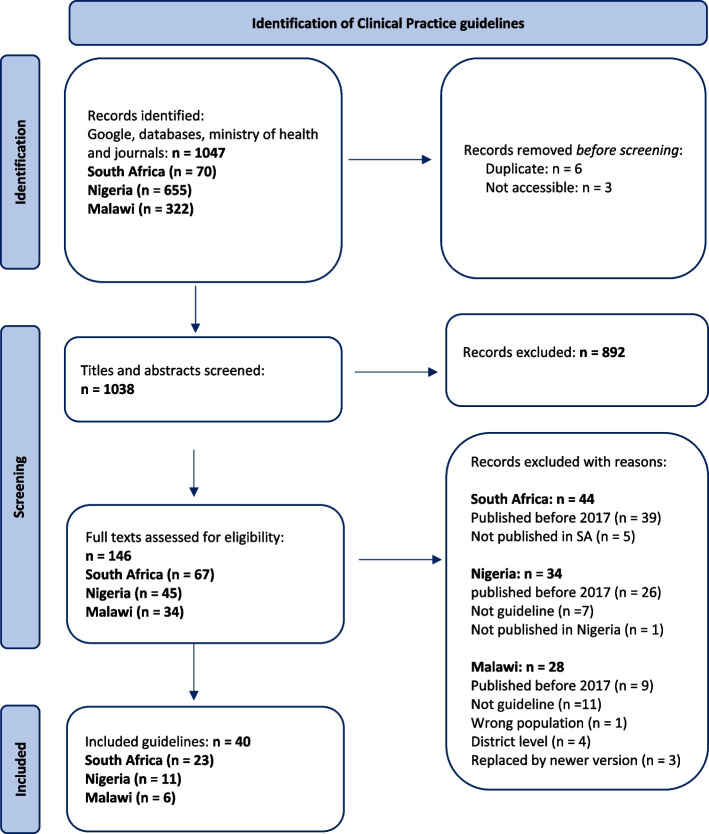
PRISMA flowchart of study selection

The searches retrieved 1047 records across the three countries, all were published in English (Fig. 1). After deduplication, we screened the titles and abstracts of 1038 records. Of these, 892 were excluded and a total of 146 potentially eligible full texts were screened. After screening, we excluded 106 records that included guidelines published before 2017 (74), international guidelines (6), focused on adults (1), irrelevant settings (4), did not fit the description of a guideline (18), and were replaced by newer versions (3). Overall, 40 guidelines met our inclusion criteria.

### Characteristics of included guidelines

We included 40 guidelines of which 58% (*n* = 23) were from South Africa, 28% (*n* = 11) from Nigeria and 15% (*n* = 6) from Malawi. Of the 40 guidelines, 20% (*n* = 8) specifically stated that they were adopted from other guidelines, and thus the remaining 80% (*n* = 32) were presumed de novo. The leading guideline developers in all three countries were ministries of health and professional associations. Of the 40 guidelines, two from South Africa were developed at the subnational level.

**Fig. 2:**
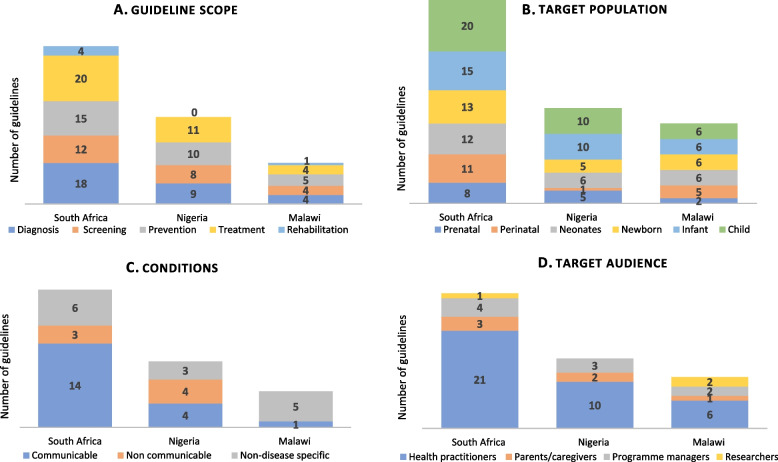
Characteristics of included guidelines

 The scope or aspects of healthcare covered by guidelines were treatment 87% (*n* = 35), prevention 75% (*n* = 30), screening 60% (*n* = 24), diagnosis 78% (*n* = 31) and rehabilitation 13% (*n* = 5) (Fig. 2A). Target populations of the guidelines were children 90% (*n* = 36) and infants 78% (*n* = 31) (Fig. 2B). Some guidelines also targeted prenatal care 38% (*n* = 15). Guidelines predominantly provided guidance on communicable diseases 48% (*n* = 19); with fewer providing guidance on non-communicable diseases (NCDs) 18% (*n* = 8). Other guidelines did not address a specific disease 35% (*n* = 14) but focused on integrated healthcare of children as well as pharmaceutical management and treatment of a wide array of diseases. These guidelines included the integrated management of childhood illnesses (IMCI) (*n* = 2), kangaroo mother care (*n* = 1), care and management of newborns (*n* = 3), and primary healthcare or hospital level standard treatment guidelines and essential medicines lists (*n* = 2) (Table 1). Across the three countries, common conditions covered by guidelines focusing on specific health issues were HIV (all countries), as well as COVID-19 and tuberculosis (Nigeria and South Africa) (Table 1). The target audience for the guidelines was mostly health professionals 93% (*n* = 37), with only a small number of guidelines considering parents / caregivers 15% (*n* = 6) as part of their target audience.

**Table 1:** Topics / conditions covered by guidelines

Conditions	South Africa (n)	Nigeria (n)	Malawi (n)
**Communicable Diseases**	**14**	**4**	**1**
HIV /AIDS	3	2	1
Tuberculosis	2	1	-
COVID-19	2	1	-
Pertussis	1	-	-
Candida Auris	1	-	-
Diphtheria	1	-	-
Listeriosis	1	-	-
Malaria	2	-	-
Pneumonia	1	-	-
**Non-Communicable diseases**	**4**	**4**	**0**
Asthma	1	-	-
Cystic fibrosis	1	-	-
Enuresis	1	-	-
Diabetes	-	1	-
Chronic pain	-	1	-
Eye health	1	1	-
Substance use disorders	-	1	-
**Other**	**6**	**3**	**5**
Primary healthcare	1	-	-
Hospital level pediatric	1	-	-
IMCI	1	-	1
Determination of death	1	-	-
Neonatal skincare	1	-	-
Cochlear implant quality standards	1	-	-
Care for infant and newborn	-	-	1
Infant and youth nutrition policy	-	-	1
Kangaroo mother care	-	1	-
Newborn management /care	-	2	1
Baby friendly hospital initiative	-	-	1

*Abbreviations:* *AIDS* Acquired immunodeficiency syndrome, *COVID-19* Coronavirus disease 2019, *HIV* Human immunodeficiency virus, *IMCI* Integrated management of childhood illnesses

Overall, 45% of the guideline (*n* = 18) described stakeholder consultation processes, with guidelines developed in Nigeria (10 of 11, 91%) and Malawi (3 of 6, 50%) showing better stakeholder involvement compared to guidelines developed in South Africa (5 of 23, 22%). Overall, systematic methods for assessing certainty of evidence were described in 18% (*n* = 7) of the guidelines, with one guideline (from South Africa) reporting use of GRADE. Guideline external review processes were described in 15% of the guidelines (*n* = 6), while methods of assessing guideline applicability (i.e., barriers and facilitators to implementation, as well as costs and resources associated with guideline implementation) were described in 13% of the guidelines (*n* = 5). About 65% of the guidelines (*n* = 26) provided a list with of guideline development group members, with Nigeria (10 of 11, 91%) and South Africa (13 of 23, 57%) showing better reporting processes for participants involved in the guideline development compared with Malawi (3 of 6, 50%).

### Guideline quality and reporting methods

**Fig. 3:**
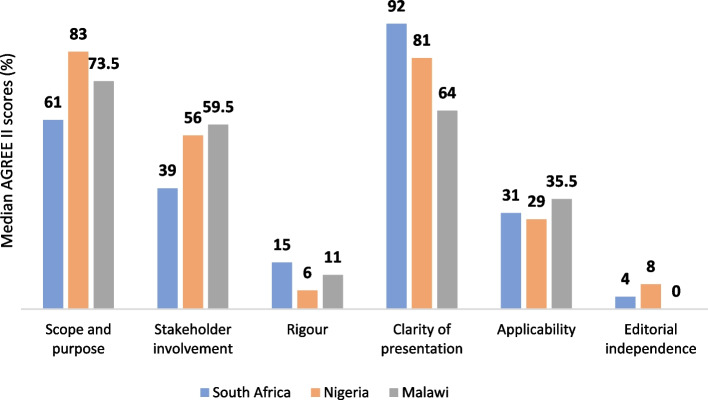
Graphical representation of AGREE II scores

The domains with the lowest median scores were editorial independence 6% (0–27), rigour of development 11% (4–32), and applicability 32% (12–50) (Table 2; Fig. 3). The domains with highest median scores were clarity of presentation 81% (67–94), and scope and purpose 68% (47–83). When stratified by country, similar trends in domain scores across guidelines from South Africa, Nigeria and Malawi were observed. Each country exhibited very low editorial independence median domain scores ranging from 0 to 8%; low rigour domain median scores ranging 6 to 15%; and low applicability domain median scores ranging from 29 to 35%.

**Table 2:** Summary of AGREE II domain scores (%) for guidelines in South Africa, Nigeria, and Malawi

Domains	Median scores (IQR)			
	South Africa *N* = 23	Nigeria *N* = 11	Malawi *N* = 6	All countries *N* = 40
**Scope and purpose**	61 (39–78)	83 (64–94)	74 (56–75)	68 (47–83)
**Stakeholder involvement**	39 (11–56)	56 (50–89)	60 (31–75)	52 (25–66)
**Rigor of development**	15 (6–38)	6 (2–26)	11 (10–38)	11 (4–32)
**Clarity of presentation**	92 (69–97)	81 (72–92)	64 (47–75)	81 (67–94)
**Applicability**	31 (6–50)	29 (17–50)	36 (27–42)	32 (12–50)
**Editorial independence**	4 (0–38)	8 (8–8)	0 (0–0)	6 (0–27)
**Overall**	42 (33–50)	33 (33–50)	50 (42–67)	42 (33–54)

## Discussion

### Summary of main results

Of the 40 guidelines included in this scoping review, most provided guidance on the management of communicable diseases, which aligns with burden of disease in the region where poverty-related infectious diseases are often associated with under-five child mortality in SSA [22]. Our main findings highlight similar issues across the three countries regarding the poor methodological quality and reporting methods of guidelines. Although most guidelines scored well for the clarity of presentation and scope and purpose domains in AGREE II, most scored very poorly on rigor of development and did not adequately describe their methods for identifying, selecting, and assessing certainty of evidence. Our findings are consistent with previous studies assessing methodological quality in both LIMC and high-income countries, where guidelines demonstrated high scores on the clarity of presentation and scope and purpose AGREE II domains, while scoring low to moderate on domains such as rigor of development, applicability, and editorial independence [23–25].

There is a lack of landscape analyses especially for guidelines addressing newborn and child health in the literature. One landscape analysis on overall South African guidelines reported on challenges with accessing guidelines, non-disclosure of funding sources and potential conflict of interests, as well as lack stakeholder consultation during guideline development [2]. The lack of appropriate methodological reporting may undermine the credibility of recommendations. Furthermore, grading the strength of recommendations and distinguishing expert opinion from evidence-based statements becomes an impossible task if appropriate links to evidence are not apparent; this is critical for transparency and guideline adaptation. Our findings further highlighted gaps in transparent reporting of funding disclosures and management of interests of members in guideline panels, as well as in considering factors such as feasibility, acceptability, and equity to inform recommendations prior to guideline implementation.

### Challenges to guideline development and adaptation in LMIC

Similar trends in guideline quality and reporting methods were observed across the three countries. This may reflect the overall challenges faced by LMICs in guideline development and implementation, including lack of resources needed, such as capacity, skills, time, and funding [26]. Using WHO guidance documents which are developed for adoption or adaptation by LMICs may therefore be a good option for these settings. However, the responsibility of appropriately assessing and considering local and contextual factors during guideline adoption / adaptation remain that of the individual countries, and the methods used for doing this are often unclear and implicit. For example, the South African and Malawian IMCI guidelines as well as the Malawian guideline on implementing the baby-friendly hospital initiative allude to the involvement of organisations such WHO, UNICEF or USAID through inclusion of their logos in the guideline documents, but without any explicit description of their role or involvement or adoption of recommendations. Scant local evidence may further complicate the guideline adoption/adaptation processes. Overall, this highlights lack of emphasis on reporting transparency with regards to guideline adaptation methods and processes to ensure that a guideline is trustworthy as well as clear linking of local evidence to recommendations. The challenges in guideline quality and reporting methods may also reflect issues with expert or stakeholder representation and the failure to include a methodologist – an expert in guideline development processes and methods in guideline development groups. The guidelines included in this review highlighted some collaborative effort between ministries of health and professional associations in the respective countries, with ministries of health being the major drivers, especially in Nigeria and Malawi. Health ministries and professional associations are often led and represented by a diverse group of front-line health professionals and clinical experts with a common goal of enhancing efficiency in resource-constrained and often understaffed health systems. Hence, guideline development and implementation may be more focused on a front-line user-centred approach with a primary goal of enhancing clarity and dissemination of recommendations for easier use, while providing little to no detail on the development process. This was reflected in the very low ‘rigor of development’ and high ‘clarity of presentation’ domain score across the three countries in our findings. Given the stark impact of financial and time constraints of developing guidelines de novo, and the heavily burdened health systems in LMICs, it may seem justifiable to front-line clinicians to publish some clinical guidance as protocols and algorithms, as reflected by documents such as the IMCI. Although most of the included guidelines provided a list of the respective guideline development groups, it was difficult to ascertain whether these groups included all relevant stakeholders as affiliations and individual roles in guideline development were often not described. For example, the observed gaps in considering health economic evidence as shown by the low scores of guidelines in the applicability domain, may potentially reflect underrepresentation of expertise such as health economists in guideline development efforts.

### Gaps in guideline topics and mismatch with disease burden

Our findings revealed potential gaps in guidelines considering locally relevant evidence when making recommendations and in addressing key conditions associated with newborn and child morbidity and mortality in those countries. For example, malnutrition, gastroenteritis and neonatal disorders contribute to the burden of disease of all three countries, yet guidance for these conditions is often included in other documents, such as the IMCI and standard treatment guidelines. In a region where poverty is often the major driver of the disease burden and child mortality and where a consistent pattern of malnutrition is observed, it would seem logical to have individual guidelines dedicated to diagnosis and management of malnutrition. Furthermore, it may be crucial to consider the emerging conditions such as NCDs, including injuries, mental health in children and preadolescents. For example, violence and intentional or unintentional injuries disproportionately affect LMICs [27], and hence guidelines for addressing injuries, trauma and associated mental health impact may be a crucial piece of the puzzle in ensuring child health and development.

### Progress in guideline methods

As countries gear up towards implementing national health insurance schemes, there has been substantial advancement in the guideline landscape in some SSA countries. For example, the standard treatment guidelines and essential medicines list are policy tools to promote rational prescribing and equitable access to cost-effective medicines in South Africa. Since the first publication of these guidelines in 1996, they have evolved to include established terms of reference for guideline committee governance, evidence review and synthesis, as well as management of interests while fostering relationships with relevant experts and methodologists in the field of evidence synthesis [28]. Furthermore, projects dedicated to strengthening capacity for development and implementation of guidelines such as the South African Guidelines Excellence and higher education on guideline training have been established [29–31].

### Limitations of the study

The lack of regional or national central guideline repositories makes it challenging to identify guideline and it is therefore possible that we may have missed relevant guidelines in the field of newborn and child health. Our search was limited to guidelines developed between 2017 and 2022; it is possible that older guidelines were missed but are still in current use or may be in the process of being updated. Despite our five-year search period, we expect that topics on most high-burden conditions should have been identified, particularly given the rate of emerging evidence for conditions such as HIV, malaria and tuberculosis requiring updated recommendations. Thus, identified gaps are likely a fair reflection of topics not adequately covered by current recommendations. Furthermore, we do not anticipate that the findings regarding overall guideline quality would have been very different in older guidelines, but rather may have been worse given the evolution of guideline reporting methods over the past decade. Some guidelines may have followed rigorous methods, but due to limited sharing of their methods in public websites or accessible repositories, this may not have been well captured in the methods. AGREE II only appraises the reporting of methods but does not cover the content of the guidelines; the recommendations may thus be appropriate even if the methods are not adequately reported.

### Implications for practice and policy

- This study suggests the importance of building skills and capacity of researchers, policy makers and development agencies that support guideline development and adaptation in Sub-Saharan Africa.
- Our results further underpin the importance of building partnerships between researchers and ministries, involving guideline methodologists where possible, when developing guidelines to ensure transparent and trustworthy guidelines,
- Guideline adaptation is likely common in LMICs, however, methods for reporting guideline adaptation need to be clearer.
- Non-disclosure of guideline funding and lack of clarity on management of potential conflict of interests may impact on credibility of recommendations.
- Guidelines that do not adequately address country-level contextual factors may face implementation challenges. For example, the unclear description of guideline panel members raises concerns about the inclusion of diverse stakeholders in decision making; and we found limited use of contextual evidence which may impact on feasibility of recommendations.

## Conclusion

There has been considerable guideline development in the three countries, with guidelines showing both strengths and weaknesses based on the AGREE II assessment. The findings of this study highlight the importance of ongoing efforts to strengthen capacity and support for guideline development. Collaboration between policymakers, researchers and all relevant stakeholders is necessary to improve and standardise guideline quality and reporting methods.

### Supplementary Information

**Additional file 1: Supplementary file (S1).** Search details.

## Data Availability

All data generated or analysed during this study are included in this manuscript (and its supplementary information files).

## Abbreviations

AGREE: Appraisal of Guidelines for Research and Evaluation
AIDS: Acquired immunodeficiency syndrome
ART: Antiretroviral therapy
COVID-19: Coronavirus 2019
GDG: Guideline Development Group
GIN: Guideline international network
GRADE: Grading of Recommendations Assessment, Development and Evaluation
HIV: Human immunodeficiency virus
IMCI: Integrated Management of Childhood Illnesses
LMIC: Lower middle-income countries
NCD: Non-communicable disease
NICE: National Institute for Health and Care Excellence
SDG: Sustainable Development Goals
SSA: Sub-Saharan Africa
UHC: Universal Health Care
WHO: World Health Organisation
