# Supplementary material for: Newborn and child health national and provincial clinical practice guidelines in South Africa, Nigeria and Malawi: a scoping review
Source: BMC Health Serv Res. 2024 Feb 19;24:221. doi: 10.1186/s12913-024-10682-0 (PMC10877834; doi:10.1186/s12913-024-10682-0)
Supplement: Supplementary file 1 — Additional file 1: Supplementary file (S1). Search details. [file 12913_2024_10682_MOESM1_ESM.docx]

**Supplementary file (S1): Search details**

**South Africa**

**Search terms**

Newborn health OR infant health OR baby health OR Perinatal health OR child health OR neonatal OR toddler health OR preadolescent child health OR Childhood illnesses OR childhood diseases

OR Infant diseases OR Newborn illnesses OR Poverty related disease OR Maternal and child

Health OR Neonat* OR Underfive* OR “under-five” OR “under-fives

**Information sources:**

Google [www.google.com](http://www.google.com)

National Department of Health <https://www.health.gov.za>

South African paediatric association <https://www.paediatrics.org.za>

Knowledge hub [www.knowledgehub.org.za](http://www.knowledgehub.org.za)

South African HIV society <https://sahivsoc.org>

South African Medical Journal [www.samj.org.za](http://www.samj.org.za)

South African Journal of HIV Medicine <http://www.scielo.org.za>

The National Institute for Communicable Diseases <https://www.nicd.ac.za>

**Nigeria**

Federal and State Ministry of Health <https://health.gov.ng>

the Paediatric Association of Nigeria (PAN) <https://pan-ng.org>

Nigerian Society of Neonatal Medicine (NISONM) <https://www.nisonm.org>

National Association of Nigeria Nurses and Midwives (NANNM) <https://nannm.com.ng>

Association of Public Health Physicians of Nigeria (APHPN) <https://aphpn-ng.org>

Society of Gynaecology and Obstetrics of Nigeria (SOGON) <https://sogon.org>

Guidelines International Network (GIN) Library [https://g-i-n.net/international- guidelines-library/](https://g-i-n.net/international-%20guidelines-library/)

Emergency Care Research Institute (ECRI) <https://www.ecri.org/>

BIGG (International Database of GRADE Guidelines) <https://sites.bvsalud.org/bigg/en/biblio/>. Journal of Paediatrics <https://www.njpaediatrics.com/>

Tropical Journal of Obstetrics and Gynaecology <https://tjog.org/index.php/tjog/>

**Malawi**

**Information sources**

National Ministry of health <https://www.malawi.gov.mw>

Ministry of Health Department <https://www.malawi.gov.mw>

Department of HIV/AIDS <https://hiv.health.gov.mw>

The Schistosomiasis Programme [www.health.gov.mw/index.php/schistosomiasis-sth-control-programme](http://www.health.gov.mw/index.php/schistosomiasis-sth-control-programme)

National Malaria Control Programme

The TB control Programme

Google [www.google.com](http://www.google.com)

Google scholar <https://scholar.google.com>
